# SPARC Expression Is Selectively Suppressed in Tumor Initiating Urospheres Isolated from As^+3^- and Cd^+2^-Transformed Human Urothelial Cells (UROtsa) Stably Transfected with SPARC

**DOI:** 10.1371/journal.pone.0147362

**Published:** 2016-01-19

**Authors:** Andrea Slusser-Nore, Jennifer L. Larson-Casey, Ruowen Zhang, Xu Dong Zhou, Seema Somji, Scott H. Garrett, Donald A. Sens, Jane R. Dunlevy

**Affiliations:** 1 Department of Pathology, School of Medicine and Health Sciences, University of North Dakota, Grand Forks, North Dakota, United States of America; 2 Department of Medicine, Division of Pulmonary, Allergy and Critical Medicine, the University of Alabama at Birmingham, Birmingham, Alabama, United States of America; 3 Department of Basic Sciences, School of Medicine and Health Sciences, University of North Dakota, Grand Forks, North Dakota, United States of America; University of Colorado School of Medicine, UNITED STATES

## Abstract

**Background:**

This laboratory previously analyzed the expression of SPARC in the parental UROtsa cells, their arsenite (As^+3^) and cadmium (Cd^+2^)-transformed cell lines, and tumor transplants generated from the transformed cells. It was demonstrated that SPARC expression was down-regulated to background levels in Cd^+2^-and As^+3^-transformed UROtsa cells and tumor transplants compared to parental cells. In the present study, the transformed cell lines were stably transfected with a SPARC expression vector to determine the effect of SPARC expression on the ability of the cells to form tumors in immune-compromised mice.

**Methods:**

Real time PCR, western blotting, immunohistochemistry, and immunofluorescence were used to define the expression of SPARC in the As^+3^-and Cd^+2^-transformed cell lines, and urospheres isolated from these cell lines, following their stable transfection with an expression vector containing the SPARC open reading frame (ORF). Transplantation of the cultured cells into immune-compromised mice by subcutaneous injection was used to assess the effect of SPARC expression on tumors generated from the above cell lines and urospheres.

**Results:**

It was shown that the As^+3^-and Cd^+2^-transformed UROtsa cells could undergo stable transfection with a SPARC expression vector and that the transfected cells expressed both SPARC mRNA and secreted protein. Tumors formed from these SPARC-transfected cells were shown to have no expression of SPARC. Urospheres isolated from cultures of the SPARC-transfected As^+3^-and Cd^+2^-transformed cell lines were shown to have only background expression of SPARC. Urospheres from both the non-transfected and SPARC-transfected cell lines were tumorigenic and thus fit the definition for a population of tumor initiating cells.

**Conclusions:**

Tumor initiating cells isolated from SPARC-transfected As^+3^-and Cd^+2^-transformed cell lines have an inherent mechanism to suppress the expression of SPARC mRNA.

## Introduction

SPARC (secreted protein, acidic and rich in cysteine) also termed osteonectin or BM-40 is a 32.5 kDa protein derived from a single copy gene which exhibits a high degree of evolutionary conservation [1]. SPARC is a matricellular protein that regulates cell-matrix interactions and tissue remodeling through the binding of collagen and other extracellular matrix proteins and through activation of matrix metalloproteinases [2, 3]. SPARC also interacts with and participates in the regulation of growth factor genes, such as, TGF-β, FGF, VEGR, and PDGF [1, 4–6]. The ability of SPARC to modulate cell-cell and cell-matrix interactions and to have de-adhesive properties has led to many studies assessing its role in tumor cell growth, differentiation, metastasis, and invasion [7–9]. The exact role that SPARC plays in the development and progression of cancer is still under investigation since SPARC has been classified as both a tumor suppressor and oncogene depending on the cancer under study. For example, low expression levels of SPARC have been demonstrated in ovarian [10] and colorectal cancer [11, 12]; whereas, high levels have been reported in breast cancer [13, 14], melanoma [15] and glioblastoma [16]. The expression of SPARC in tumor stroma has been associated with a poor prognosis in non-small cell lung cancer [17] and with disease recurrence in breast ductal carcinoma *in situ* [18]. Low expression of SPARC in stroma predicted a poor prognosis for patients with colon cancer [19].

This laboratory’s interest in SPARC expression is the role it might have in the development and progression of urothelial cancer in general and in environmental-induced urothelial cancer in particular. SPARC has been shown to be expressed at the luminal surface of normal human urothelium [20] and primary cultures of human urothelial cells have been shown to both express SPARC and to secrete SPARC into the conditioned growth medium [20, 21]. The level of SPARC mRNA has been shown to correlate with increased histological grade, pathological stage, and poor prognosis in urothelial cancer; however, the expression of SPARC protein was not determined in this study [22]. In a recent study using transgenic mice lacking SPARC expression, it was shown that the loss of SPARC expression correlated with an increase in the development and progression of urothelial cancer [23]. The development of bladder cancer is known to have a strong association with environmental exposures [24] and this laboratory employs the UROtsa cell line as a model to explore the relationship between As^+3^ and Cd^+2^ exposure and the development of urothelial cancer. The UROtsa cell line is an immortalized, non-tumorigenic model that retains features of transitional urothelium when propagated using a serum-free growth medium [25, 26]. This cell line has been used to show that both Cd^+2^ and As^+3^ can cause the malignant transformation of human urothelial cells [28–30]. These resulting As^+3^- and Cd^+2^-transformed cell lines were all shown to retain a morphology consistent with human urothelial cancer and to display phenotypic differences characteristic of tumor heterogeneity. The histology of subcutaneous tumor heterotransplants produced by these transformed isolates displayed histologic features of human urothelial carcinoma with areas of squamous differentiation. This observation is important since urothelial carcinoma is the most prominent type of bladder cancer in western countries and accounts for over 95% of all cases and is 5^th^ in overall occurrence [24].

A previous study from this laboratory analyzed the expression of SPARC in the parental UROtsa cells, their As^+3^- and Cd^+2^-transformed cell lines, and the resulting tumors when the transformed cells were transplanted subcutaneously in immune compromised mice [31]. It was demonstrated that SPARC expression was down-regulated to background levels in Cd^+2^- and As^+3^-transformed UROtsa cells compared to parental cells. In addition, it was shown by tumor transplantation into immune-compromised mice that the malignant epithelial component of tumors derived from these cell lines was also down-regulated to background levels for SPARC expression, but that the stromal cells recruited to these tumors was highly reactive for SPARC. This finding was shown to translate to specimens of human urotheial cancer where tumor cells were SPARC negative, but stromal cells were positive. It was also shown that acute exposure of the parental UROtsa cells to either As^+3^ or Cd^+2^ resulted in a marked reduction in SPARC expression. The finding that the As^+3^-and Cd^+2^-transformed cell lines had only background expression of SPARC offered the opportunity to re-introduce SPARC expression back into these cell lines. In the present study, the As^+3^-and Cd^+2^-transformed cell lines were stably transfected with a SPARC expression vector, and the effect of SPARC expression on the ability of the cells to form tumors in immune compromised mice and on *in vitro* cell growth, migration, and invasion was determined.

## Materials and Methods

### Cell Culture

The UROtsa cell line was initially developed and characterized by Petzoldt [25], and further developed and characterized in this laboratory [26]. All available UROtsa cells within the scientific community have originated directly or indirectly from this laboratory. Although it is stated by Johnen [27] and in the database of contaminated cell lines (ICLAC Database of Cross-contaminated or Misidentified Cell Lines) that the UROtsa cell line from some sources have been contaminated by the human bladder cancer cell line, T24, this laboratory has a history of never having cultured T24. Thus the contamination event would have occurred through secondary sources after the UROtsa cell line was given to other investigators. Short tandom repeat analysis (STR) of the UROtsa cell line shown in the table below does not match any cell line in the ATCC STR database indicating that it is a unique cell line and certainly does not contain any T24 cells (S1 Table). Mycoplasma testing by PCR- and enzymatic-based methods indicates that this cell line is mycoplasma free. Stock cultures of the parental UROtsa cell line were maintained in 75 cm^2^ tissue culture flasks using Dulbecco’s modified Eagle’s medium (DMEM) containing 5% v/v fetal calf serum in a 37°C, 5% CO_2_: 95% air atmosphere [26]. The isolation and growth of the 7 isolates of the Cd^+2^-transformed isolates and the 6 individual isolates of the As^+3^-transformed UROtsa cells has been described previously [28–30]. Two As^+3^ and 2 Cd^+2^-transformed cell lines were used in the present study, As#3, As#6, Cd#1 and Cd#4, respectively. The transformed cell lines were all grown and maintained using identical conditions. Briefly, confluent flasks were sub-cultured at a 1:4 ratio using trypsin-EDTA (0.05%, 0.02%) and the cells were fed fresh growth medium every 3 days. The MDA-MB-231 and Hs578T cell lines were obtained from the American Type Culture Collection (ATCC) and grown following the ATCC-supplied protocols. Cell viability and doubling times were determined by measuring the capacity of the cells to reduce MTT (3-(4,5-dimethylthiazol-2-yl)-2,5-diphenyltetrazolium bromide) to formazan. Triplicate cultures were analyzed for each time point and concentration.

### Stable Transfection of the As^+3^ and Cd^+2^ Transformed Cell Lines

The SPARC ORF was cloned into the pENTR221 vector and transferred into the destination vector, pcDNA6.2/V5-DEST by the LR recombination reaction (Invitrogen). The purified plasmid constructs were verified by sequencing. The DNA constructs were linearized prior to transfection. The selected As^+3^-and Cd^+2^-transformed UROtsa cell lines were transfected with the SPARC ORF (S2 Table) in pcDNA6.2/V5-DEST or the blank vector using the Effectene Transfection reagent (Qiagen) at a 1:10 plasmid to Effectene ratio. The lipid complexes were added to the cells at 2 μg of DNA per 9.6 cm^2^ culture well. Clones were selected using cloning rings and propagated in growth media containing 4 μg/ml Blasticidin (Invitrogen). Multiple clones from each cell line were analyzed to confirm SPARC expression and one clone from each line selected for further study. A blank vector control for each cell line was also selected for study.

### Assessment of Vector Copy Number

Genomic DNA was isolated and purified from the transfected cell lines using DNeasy Blood and Tissue Kit (Qiagen, Valencia, CA) according to the manufacturer’s instructions. Approximately 5 x 10^6^ cells were pelleted, and resuspended in PBS prior to the addition of proteinase K. To prevent carryover of RNA, the suspension was incubated with 1.8 μg/μL RNase A for 2 minutes at room temperature. An equal volume of lysis buffer was added to the cell suspension, mixed, and incubated at 56°C for 10 minutes. Ethanol was added to the suspension and mixed thoroughly before loading onto a DNeasy mini spin column. The bound DNA was washed 3 times prior to elution with 10 mM Tris-HCl, pH 7.5. For qPCR of the genomic DNA, the same conditions were used as that for qRT-PCR, except 100 ng of input DNA was used rather than 10 ng. Cycling parameters were 15 sec denaturation at 95°C, 30 sec annealing at 58°C, 30 sec extension at 72°C. The SPARC expression plasmid, SPARC-pcDNA6.2/V5-DEST, was used as a quantitative standard for the PCR reaction.

### Preparation of Spheroids (Urospheres) from Cultures of the As^+3^-and Cd^+2^-Transformed Cell Lines Stably Transfected with SPARC and a Blank Vector Control

For the formation of urospheres, the SPARC transformed cell lines and the blank vector controls were seeded at a density of 10^5^ cells in T-25 cm^2^ Ultra-low attachment flasks (Corning Inc., Corning NY). The growth medium consisted of 1:1 mixture of Dulbecco’s modified Eagles’ medium and Hams’s F-12 growth medium supplemented with selenium (5 ng/ml), insulin (5 μg/ml), transferrin (5 μg/ml), hydrocortisone (36 ng/ml), triiodothyronine (4 pg/ml), and epidermal growth factor (10 ng/ml). The cells were allowed to form spheres for 8 days after which the urospheres were harvested by centrifugation and were used for RNA isolation or tumor transplantation into immune compromised mice.

### Tumor Transplantation into Immune Compromised Mice

All four transformed cell lines, corresponding SPARC-transfected (and blank vector-transfected) cell lines, and derived urospheres were capable of producing tumors when inoculated subcutaneously into immune compromised mice. The As#3 and Cd#1 lines also formed tumors when injected into the peritoneal cavity of immune compromised mice. The protocol for the transplantation of the transformed UROtsa cell lines into immune compromised mice has been described previously [29]. Briefly, five nude (NCr-nu/nu) mice (per cell line or urosphere) were inoculated subcutaneously in the dorsal thoracic midline with 1 x 10^6^ cells. All mice were sacrificed by 10 weeks post-injection or when clinical conditions dictated euthanasia. The maximum tumor size was 2.5 cm. Tumor samples were either paraffin-embedded, sectioned, stained with hemotoxylin and eosin (H&E) and analyzed by light microscopy or snap frozen and processed for RNA and protein isolation. An identical procedure was used for the subcutaneous transplantation of the SPARC-transfected cell lines, blank vector (DEST) cell lines, and urospheres.

### Ethics Statement

This study adhered to all recommendations dictated in the Guide for the Care and Use of Laboratory Animals of the NIH. The specific protocol was approved by the University of North Dakota Animal Care Committee (IACUC #1110-2C). All efforts were taken in order to minimize animal suffering, and mice were euthanized when clinical conditions dictated. Animals were sacrificed by 10 weeks post tumor transplantation by CO_2_ inhalation and euthanasia conformed to AVMA Guidelines on Euthanasia.

### Expression and Localization of SPARC in UROtsa Cells and Tumor Transplants

The preparation of total RNA and protein from the parental UROtsa cell line and from the Cd^+2^-and As^+3^-transformed cell lines and their subcutaneous transplants have been described previously [28–30]. The expression and localization of SPARC mRNA and protein in cell lines by real-time quantitative reverse transcription polymerase chain reaction (qRT-PCR), western blotting, and immunofluorescence has been described previously [31]. The expression and localization of SPARC in tumor transplants by real time RT-PCR, western blotting, and immunohistochemical localization has also been described previously [31]. See S2 Table for primer information.

### Secretion of SPARC into the Culture Medium

The expression of secreted SPARC protein was determined by western analysis using slight modification in the protocol as previously described by Sage [17]. Briefly, conditioned media from confluent cultures was collected at 24 h and 48 h, centrifuged, and filtered through a 0.22 μm filter. Solid ultrapure ammonium sulfate (Sigma Aldrich, St. Louis, MI) was gradually added to 50% w/v of starting conditioned media volume over several hours with mixing at 4°C. Media was then precipitated for 16 h at 4°C with end-on-end rotation followed by centrifugation at 40,000 x g. The supernatant was discarded and the resulting pellet was dissolved in 2% SDS buffer for analysis. Equal amounts (20 μg) of total protein were loaded.

### Migration Assay

The migration potential of parental UROtsa cells, the As^+3^-and Cd^+2^-transformed cells, the SPARC and DEST-transfected UROtsa cells and the breast cancer cell line MDA-MB-231 (positive control) were assessed. Analysis of migration was conducted using two assays, a wound/scratch assay and a trans-well chemotaxis migration assay. For the wound/scratch assay, cells were grown to confluence and treated with Mitomycin C (MMC) (Sigma Aldrich) for 2 h, to inhibit cellular division. Appropriate concentrations of MMC were specifically determined for each cell line by MTT analysis to insure inhibition of cellular proliferation while also insuring that levels were not toxic. A scratch was made within the cell monolayer using a sterile 200 μL pipette tip. The monolayer was then washed with phosphate-buffered saline, fresh growth medium was added, and cells were allowed to migrate for 24 or 48 h. Cells were photographed by light microscopy (using a 10X magnification lens) at 0, 24, and 48 h to analyze the migration of the cells towards the “wounded” area.

Analysis of migration by chemotaxis was conducted using 24-well trans-well inserts with an 8 μm pore size polycarbonate membrane (Cell Biolabs, San Diego, CA). 7.2 x10^4^ cells in 300 μL of serum free media was added to the upper chamber and 500 μL of media containing 1.5% fetal calf serum was added to the bottom chamber of each well. The cells were allowed to migrate for 8 h at 37°C, 5% CO_2_: 95% air atmosphere following which they were fixed and stained with the supplied solutions and total cells were counted using light microscopy. After counting the total number of cells, the non-migratory cells were gently swabbed off the top insert membrane and the remaining migratory cells on the bottom side of the membrane were counted. Twenty fields were per insert was counted for each side of the membrane and the percentage of migrated cells was determined.

### Invasion Assay

The invasion potential of the UROtsa parent cells, the As^+3^-and Cd^+2^-transformed cells, the SPARC and DEST-transfected UROtsa cells and the breast cancer cell line Hs578t (positive control) was assessed using basement membrane coated 24-well trans-well inserts. Cells were pretreated with MMC for 2 h before they were trypsinized and added to the upper chamber of the basement membrane coated 8 μm pore size polycarbonate membrane (Cell Biolabs) insert. The same concentrations of MMC were used as in the wound assay. 7.2 x10^4^ cells in 300 μL serum free media was added to the upper chamber and 500 μL of media containing 10% fetal calf serum was added to the bottom chamber of each well. The cells were allowed to invade for 24 h at 37°C, 5% CO_2_: 95% air atmosphere. Cells were stained and counted in the same manner as the chemotaxis migration assay. The assay was performed in duplicate and the percentage of cells that invaded was determined.

### Expression of ALDH1a1 in UROtsa Cell Lines and Spheroids (Urospheres)

The expression of ALDH1a1 mRNA was assessed by qRT-PCR. Purified RNA (100 ng) was subjected to cDNA synthesis using the iScript cDNA synthesis kit (Bio-Rad Laboratories, Hercules CA) in a total volume of 20 μl. Real-time PCR was performed utilizing the SYBR Green kit (Bio-Rad Laboratories) with 2 μl of cDNA, 0.2 μM commercially available primers (Qiagen) in a total volume of 20 μl. Amplification was monitored by SYBR Green fluorescence in a CFX96 Touch^™^ Real-Time Detection System (Bio-Rad Laboratories). Cycling parameters consisted of denaturation at 95°C for 15 seconds, annealing at 62°C for 30 seconds, and extension at 72°C for 30 seconds which gave optimal amplification efficiency. The expression level of ALDH1a1 was normalized to levels of 18S expression and is expressed in (mean ± SEM) transcripts ALDH1a1/10^6^ transcripts 18S.

### Statistical Analysis

All experiments were performed in triplicate unless otherwise stated and the results are expressed as the mean ± SEM. Statistical analyses were performed using GraphPad Prism^®^ software using separate variance t-tests or ANOVA with Tukey post-hoc testing. Unless otherwise stated, the level of significance was 0.05.

## Results

### SPARC mRNA and Protein Expression in Stably Transfected As^+3^-and Cd^+2^-Transformed UROtsa Cell Lines

**Fig 1:**
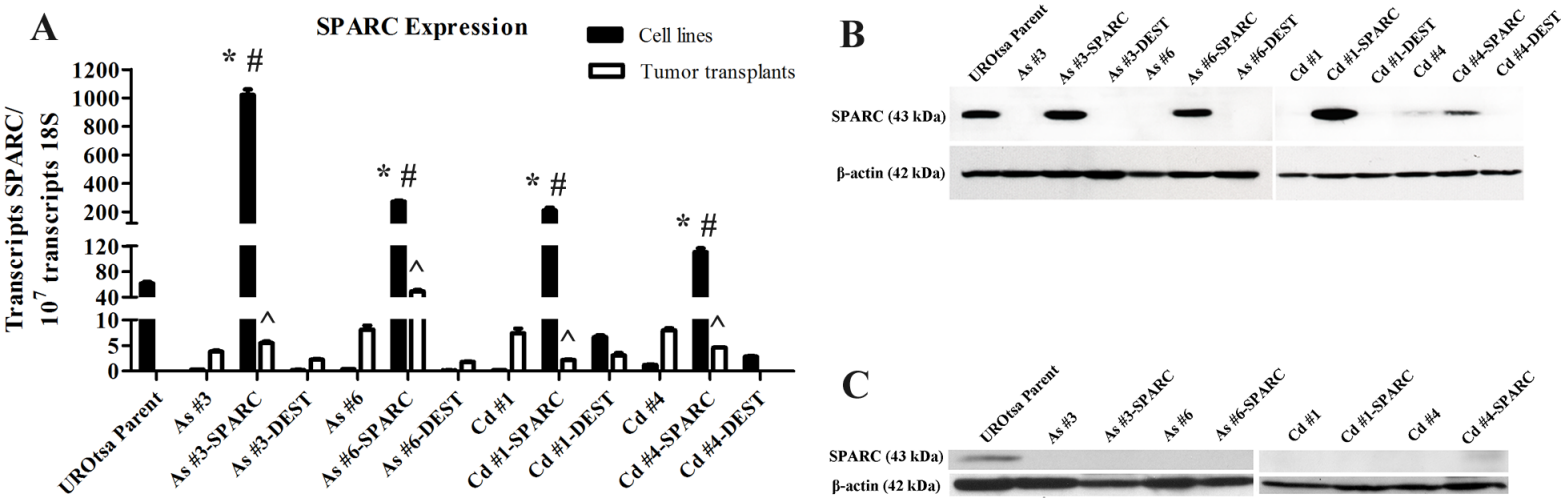
Expression of SPARC mRNA and protein in parental UROtsa, As^+3^ or Cd^+2^ transformed UROtsa, blank vector (DEST)-transfected As^+3^ or Cd^+2^ transformed UROtsa SPARC-transfected As^+3^ or Cd^+2^ transformed UROtsa cell lines and corresponding tumor transplants. (A) Real time-RT-PCR analysis of SPARC expression. The resulting mRNA levels were normalized to the number of 18S transcripts. Real time data is plotted as the mean ± SEM of triplicate determinations; * denotes significant increase compared to UROtsa (p<0.05); # denotes significant increase compared to non-transfected transformed cell line (p<0.05); ^ denotes significant decrease compared to the corresponding SPARC-transfected cell line (p<0.05). (B) Western analysis of SPARC protein expression (stripped and reprobed for β-actin) in the cell lines and (C) corresponding tumor transplants. The #’s identify the independent cell lines isolated by the exposure of UROtsa cells to As^+3^ or Cd^+2^ as described by Cao et al. 2010 [29] and Somji et al. 2010 [30], respectively.

Two As^+3^ (#3 and #6) and 2 Cd^+2^ (#1 and #4) transformed UROtsa cell lines were stably transfected with the SPARC ORF under the control of the CMV promoter. An analysis of SPARC mRNA expression confirmed that the As^+3^-and Cd^+2^-transformed cells had significantly reduced levels of SPARC mRNA compared to the parental UROtsa cells (Fig 1A). The parental UROtsa cells contained approximately 100 transcripts of SPARC mRNA per 10^7^ transcripts of 18S ribosomal RNA; whereas, the As^+3^-and Cd^+2^-transformed cells possessed 0.3 to 1.0 transcripts of SPARC mRNA per 10^7^ transcripts of 18S ribosomal RNA. This level of SPARC expression would be indicative of near background levels of SPARC mRNA in the As^+3^-and Cd^+2^-transformed cells prior to stable transfection with a SPARC-containing expression vector (Fig 1A). The stable transfection of the As^+3^-and Cd^+2^-transformed cell lines with the SPARC ORF resulted in an increased expression of SPARC mRNA in each of the 4 transformed cell lines (Fig 1A). All four of these cell lines had levels of SPARC mRNA expression exceeding that of the parental UROtsa cells, ranging from 1 to 4 fold higher (Fig 1A). The stable transfection of the As^+3^-and Cd^+2^-transformed cell lines with the expression vector without the SPARC ORF were shown to have SPARC mRNA levels that were unchanged compared to those of the original non-transfected As^+3^-and Cd^+2^-transformed cell lines (Fig 1A).

**Fig 2:**
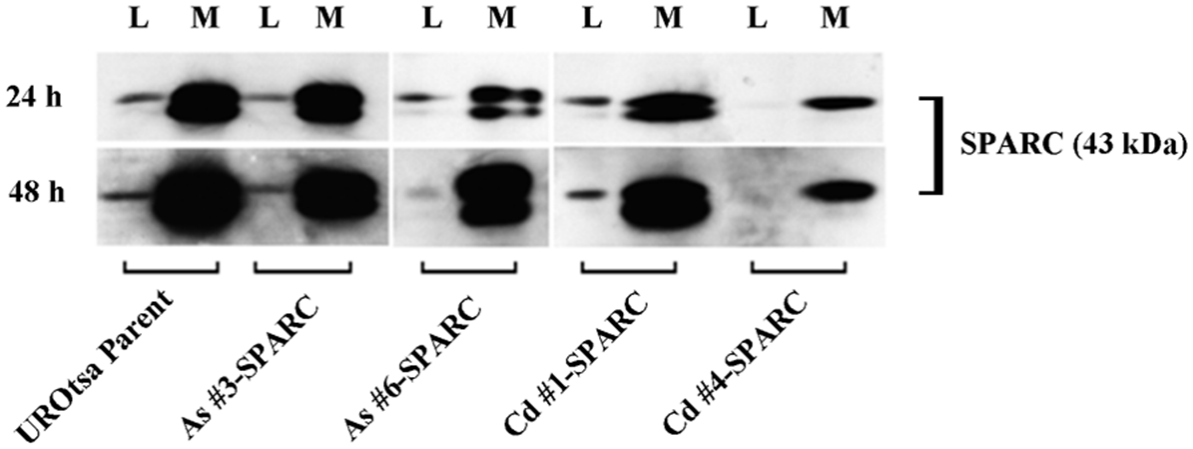
Secretion of SPARC into culture media. Western analysis of SPARC protein secreted from confluent cultures of the parental UROtsa cells and SPARC-transfected cells. Conditioned media (M) was collected at 24 h (top panel) and 48 h (bottom panel) time points then concentrated as described in materials and methods and was compared to their respective cell lysates (L).

A corresponding analysis of SPARC protein expression in lysates prepared from the above cell lines showed that only the parental UROtsa cells and the SPARC transfected As^+3^-and Cd^+2^-transformed cell lines expressed the SPARC protein (Fig 1B). The expression of the SPARC protein was similar among the parental UROtsa cells and 3 of the 4 SPARC-transfected cell lines (As#3, As#6, Cd#1). One SPARC-transfected cell line (Cd#4) displayed a reduced level of the SPARC protein when compared to the other SPARC-transfected cell lines. Since SPARC is known to be a protein that is generally secreted by cells, the relative level of SPARC protein secreted into the culture medium was determined for the parental UROtsa cells and the SPARC transfected As^+3^-and Cd^+2^-transformed UROtsa cells. The level of SPARC protein released into the culture medium was determined 24 and 48 h after switching confluent cultures of cells from a serum-containing to a serum-free cell culture condition. This analysis showed that the parental UROtsa cell line did secrete SPARC into the medium and that secretion of SPARC increased between 24 and 48 h (Fig 2). This analysis demonstrated that each of the SPARC transfected As^+3^-and Cd^+2^-transformed cell lines also secreted SPARC protein into the culture medium with levels increasing between 24 and 48 h of culture. The Cd#4-SPARC transfected cell line that showed lower SPARC protein expression in cell lysates compared to the other transfected lines, also had less SPARC protein secreted into the medium. Overall, the parental UROtsa cells and the SPARC transfected As^+3^-and Cd^+2^-transformed cell lines all expressed SPARC mRNA and protein with secretion of SPARC into the culture medium.

### SPARC Expression in Tumor Transplants Generated from the SPARC-Transfected As^+3^-and Cd^+2^-Transformed Cell Lines

Tumor transplants were generated from the SPARC-transfected As^+3^-and Cd^+2^-transformed lines and their counterparts transfected with the expression vector without the SPARC ORF. Four mice were inoculated subcutaneously with 5 x 10^6^ cells for each of the above cell lines. The growth of the SPARC-transfected and vector control tumor transplants in the immune compromised mice were similar to each another (data not shown) and to that previously published for the original non-transfected transformed cell lines [28–30]. Growth was judged as a qualitative measure of the time interval between injection and harvest of the tumor (tumors are harvested between 1 and 2 cm in diameter). Samples from each tumor were prepared for the analysis of mRNA expression by real time RT-PCR and protein expression by both western blotting and immunohistochemistry. The results of this analysis demonstrated that the level of SPARC mRNA expression in the tumor transplants generated from 3 of the 4 SPARC transfected As^+3^ and Cd^+2^ transformed cell lines (As#3, Cd#1, Cd#4) was significantly reduced when compared to both the SPARC-transfected cell line and to levels present in the parental UROtsa cells (Fig 1A). The levels of SPARC mRNA in the tumors from these 3 SPARC-transfected cell lines was similar to that found in tumors generated from the transformed cell lines not transfected with SPARC and from tumors generated from the transformed cell lines containing a vector control without the SPARC ORF (Fig 1A). The only tumor transplant having a level of SPARC mRNA expression convincingly above background was the As#6 cell line transfected with SPARC (Fig 1A). The tumor from this cell line displayed SPARC mRNA expression below that present in the corresponding SPARC-transfected cell line and parental UROtsa cells, but clearly above the background levels present in tumors generated from the As#6 cell line with and without the vector control (Fig 1A). Western blotting of lysates compared for the tumor transplants generated from the SPARC-transfected As^+3^-and Cd^+2^-transformed cell lines demonstrated no expression of the SPARC protein for the tumors generated from the As#3, As#6, and Cd#1 cell lines transfected with SPARC (Fig 1C). The lysate prepared from the tumor generated from the Cd#4 cell line transfected with SPARC did demonstrate a faint band representative of the SPARC protein. The tumors generated from the 4 transformed cell lines transfected with the vector control were negative for the expression of the SPARC protein (data not shown).

**Fig 3:**
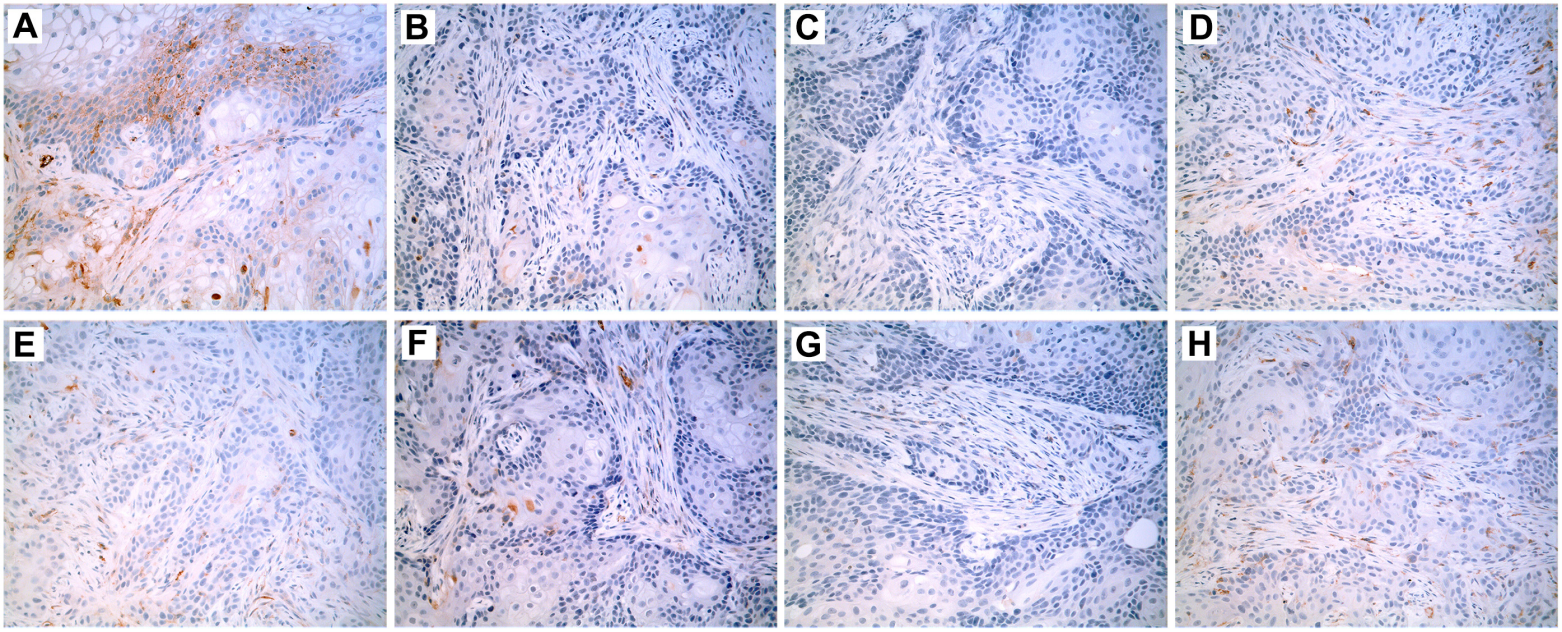
Immunohistochemistry of SPARC localization in tumor transplants generated from SPARC-transfected cell lines. Immunohistochemical stain (brown) shows the location of SPARC expression within the tumor transplant tissues generated from the following UROtsa cell lines: (A) As#3-SPARC; (B) As#3-DEST; (C) As#6-SPARC; (D) As#6-DEST; (E) Cd#1-SPARC; (F) Cd#1-DEST; (G) Cd#4-SPARC; and (H) Cd#4-DEST cell lines. All images are shown with a 200X magnification.

Immunostaining for the SPARC protein, which is a technique capable of identifying SPARC protein expression in small, focal areas of a tumor, was performed on formalin fixed, paraffin embedded samples of the tumor transplants generated from the SPARC transfected As^+3^ and Cd^+2^ cell lines (Fig 3). The results of this analysis demonstrated no expression of SPARC in the tumor transplants generated from the As#6, and Cd#4 cell lines stably transfected with the SPARC open reading frame (Fig 3C and 3G), whereas Cd#1 exhibitied trace focal expression (Fig 3E). In contrast, the As#3 cell line transfected with the SPARC open reading frame demonstrated multiple areas of focal expression of the SPARC protein representative of approximately 10% of the overall tumor mass (Fig 3A). All four As^+3^ and Cd^+2^ transformed cell lines transfected with the blank vector without the SPARC ORF showed no immunoreactivity for the SPARC protein (Fig 3B, 3D, 3F and 3H). This finding is in agreement with the previous study from this laboratory which showed that tumor transplants derived from the non-transfected As^+3^-and Cd^+2^-transformed cell lines were all negative for SPARC immunostaining [31]. The antibody against the SPARC protein used for immunostaining in the present study has been shown to have greater specificity for human SPARC than for mouse SPARC when compared to the one used by this laboratory in the previous study.

### Tumor Transplants Generated from Urospheres Isolated from As^+3^-and Cd^+2^-Transformed UROtsa Cells Stably Transfected with SPARC

**Fig 4:**
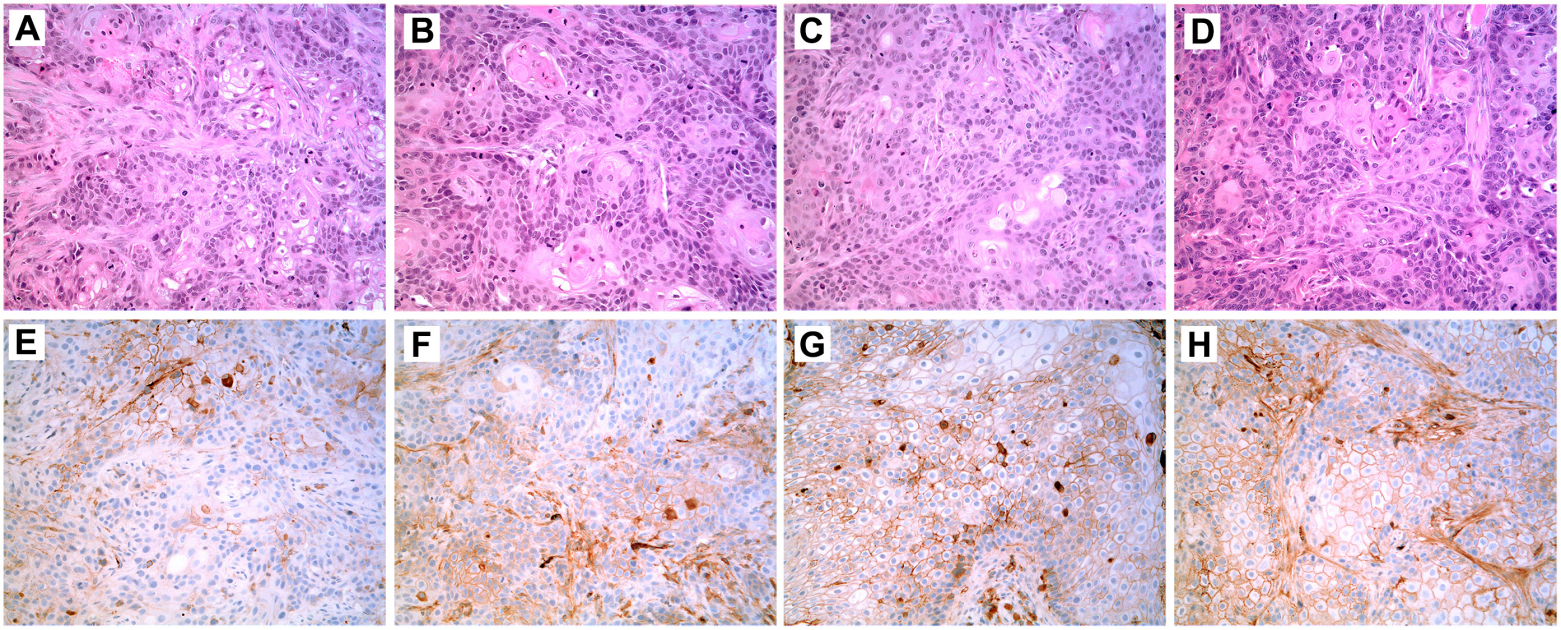
Histology and Immunohistochemistry of SPARC in Tumor Transplants Generated from Urospheres Isolated from As^+3^-and Cd^+2^-Transformed UROtsa Cell Lines and from As^+3^-and Cd^+2^-Transformed UROtsa Cell Lines Stably Transfected with SPARC. Tumor transplants were generated from urospheres isolated from four transformed UROtsa cell lines: As#3 (A, E); As#3-SPARC (B, F); Cd#4 (C, G); and Cd#4-SPARC (D, H). The histology of the tumors generated from the urospheres is shown in (A-D) while the SPARC immunolocalization (brown stain) is shown in (E-H). All images are shown with a 200X magnification.

Urospheres, potentially representing a population of tumor initiating cells (TIC), were isolated from the As^+3^-and Cd^+2^-transformed UROtsa cell lines stably transfected with SPARC. Urospheres were also isolated from the As^+3^-and Cd^+2^-transformed UROtsa cell lines that were not transfected with SPARC and were used as a control. The ability of the urospheres to produce a subcutaneous tumor in immune compromised mice was used to judge their ability to be defined as a population of TIC. The results demonstrated that the urospheres isolated from the As^+3^-and Cd^+2^-transformed UROtsa cell lines not transfected with SPARC were able to form a subcutaneous tumor when transplanted into immune compromised mice. The histology of the tumors produced from the urospheres was similar to one another and consistent with urothelial cancer with areas of squamous differentiation (Fig 4A and 4B, illustrated for As#3 and Cd#4). The histology of the tumors produced from the urospheres were identical to the tumors produced by the As^+3^-and Cd^+2^-transformed UROtsa cell lines as published previously by this laboratory [28–30]. The finding that the urospheres were able to produce tumors in immune compromised mice allows them to be defined as a population of TIC. An identical determination was performed for the As^+3^-and Cd^+2^- transformed UROtsa cell lines transfected with SPARC and each were able to form a subcutaneous tumor when transplanted into immune compromised mice (Fig 4C and 4D, illustrated for As#3 and Cd#4). The histology of the tumors produced from the SPARC-transfected urospheres were identical to the tumors produced by the non-SPARC transfected urospheres and with that of the As^+3^- and Cd^+2^-transformed UROtsa cell lines as published previously by this laboratory [28–30]. The ability of the SPARC-transfected urospheres to generate a subcutaneous tumor in immune compromised mice allows them to be defined as a population of TIC.

### Expression of ALDH1A1 in non-transfected and SPARC-Transfected As^+3^-and Cd^+2^-transformed UROtsa Cell Lines and Urospheres

**Fig 5:**
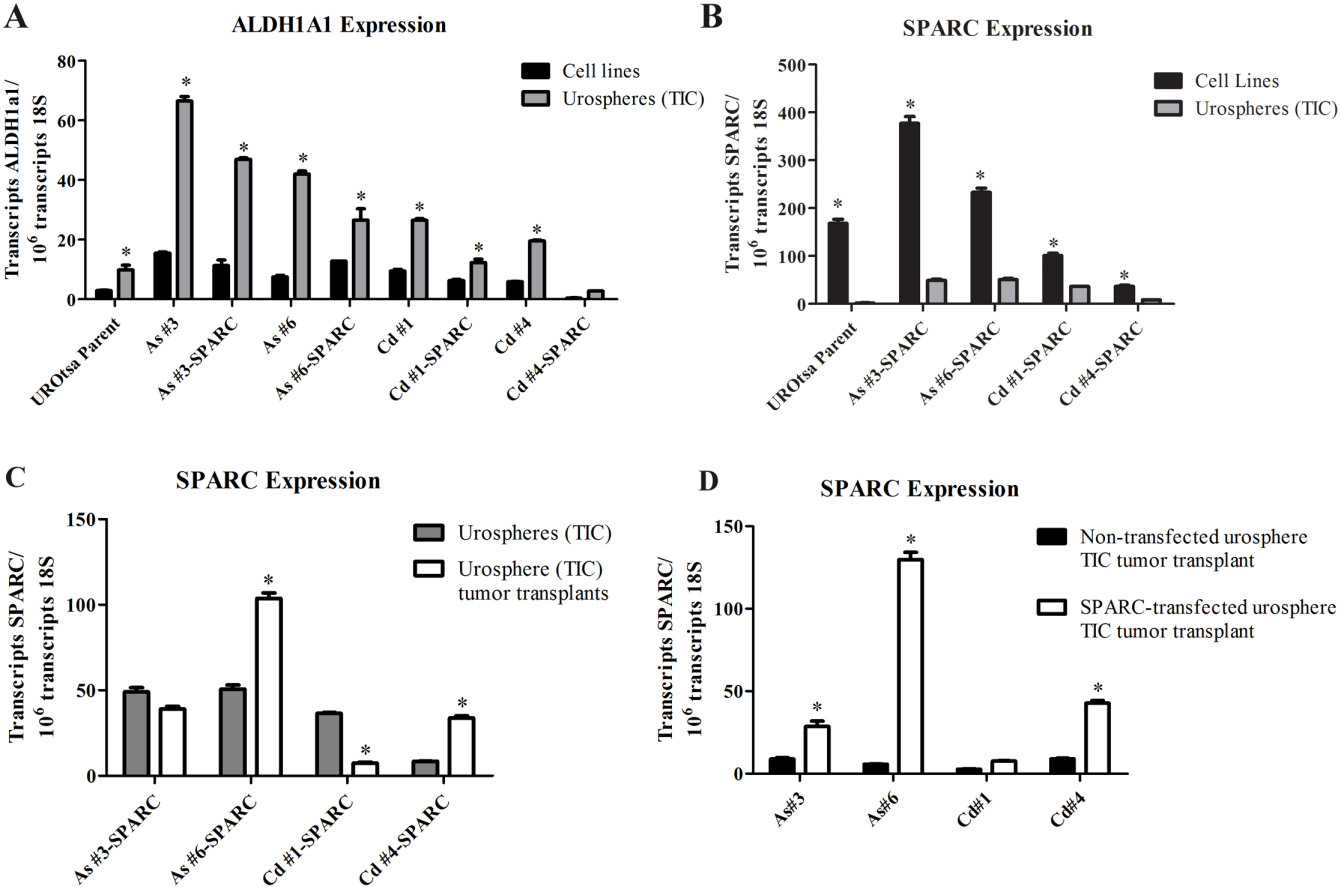
Expression of ALDH1A1 and SPARC mRNA in urospheres (TIC) and SPARC in TIC tumor transplants. Real time RT-PCR was used to compare mRNA expression of ALDH1A1 (A) and SPARC (B) in the non-transfected and SPARC transfected UROtsa transformed cell lines (grown in serum free conditions) to the corresponding urospheres (TIC) generated from these cultures. SPARC mRNA expression was also quantitated from TIC tumor transplant tissue generated from the urospheres (TIC). Levels of SPARC mRNA were then compared between the SPARC-transfected urospheres and the corresponding SPARC-transfected urosphere (TIC) tumor transplants (C) and between the non-transfected urosphere (TIC) tumor transplants and the SPARC-transfected tumor transplants (D). Results are expressed as transcripts ALDH1A1/or SPARC per 10^6^ transcripts of 18S. *Indicates significant difference (p<0.05) from corresponding cell line (A), urosphere (B-C), or non-transfected TIC tumor (D).

The expression of ALDH1A1 mRNA, a TIC marker, was determined using total RNA isolated from the TIC from the SPARC-transfected and non-transfected As^+3^-and Cd^+2^-transformed UROtsa cell lines. The results of this determination showed that ALDH1A1 mRNA was significantly enriched in TIC from both the SPARC-transfected and non-transfected As^+3^-and Cd^+2^-transformed UROtsa cell lines (Fig 5A). The level of ALDH1A1 was variable among the cell lines and could give some indication of the level of TIC in each line, if one were to assume the level of ALDH1A1 is proportional to the extent of the TIC population.

### Expression of SPARC mRNA in Urospheres (TIC) and Corresponding Tumor Transplants from SPARC-Transfected As^+3^-and Cd^+2^-transformed UROtsa Cell Lines

The expression of SPARC mRNA was determined using total RNA isolated from the urospheres (TIC) derived from the SPARC-transfected As^+3^-and Cd^+2^-transformed UROtsa cell lines. The results of this analysis demonstrated that SPARC mRNA was reduced to a background level of expression in the urospheres (TIC) isolated from all four SPARC-transfected As^+3^-and Cd^+2^-transformed UROtsa cell lines (Fig 5B). This is in marked contrast to the much higher levels of SPARC mRNA present in the SPARC-transfected cell lines that served as the parent culture for the isolation of the urospheres (TIC) (Fig 5B). The level of SPARC mRNA was also determined on total RNA prepared from the tumor transplants generated by the TIC from the SPARC-transfected As^+3^-and Cd^+2^-transformed cell lines (TIC tumor transplants). In contrast to the consistently observed low levels of SPARC found in the SPARC-transfected urosphere (TIC) cultures, the level of SPARC mRNA in the SPARC-transfected urosphere (TIC) generated tumor transplants was variable (Fig 5C). In particular, As#6-SPARC displayed significantly higher levels of SPARC mRNA (Fig 5C and 5D) and approached levels of the UROtsa parental cell line (Fig 5B). Additionally, in contrast to the low levels found in the non-transfected urosphere (TIC) tumor transplants, the SPARC-transfected urosphere (TIC) tumor transplants had increased levels of SPARC in 3 of the 4 TIC tumors (Fig 5D). In order to confirm that the decreased levels of SPARC mRNA and protein were not due to the loss of the vector, we analyzed genomic DNA from each of the SPARC-transfected cell lines and TIC for the presence of BSD vector sequence (S3 Table). The results of this analysis confirmed the construct was present and stably integrated in all SPARC-transfected cell lines and TIC used for tumor transplantation

### SPARC Protein Expression in Tumor Transplants Generated from Urospheres (TIC)

Western blotting of protein lysates prepared from tumors produced by the urospheres (TIC) isolated from all 4 SPARC-transfected and non-SPARC transfected cell lines all showed no expression of the SPARC protein (data not shown). Immunohistochemistry was used to determine if the SPARC protein might have focal expression in the tumors generated from the urospheres (TIC) generated from the 4 SPARC-transfected and non-SPARC transfected cell lines. Immunolocalization using a SPARC antibody demonstrated that 3 of the 4 TIC tumor transplants (As#3, As#6, Cd#4) generated from the non-SPARC transfected urospheres (TIC) had focal expression of the SPARC protein representing between 10 to 20% of the total tumor mass (Fig 4E and 4F, focal staining illustrated for As#3 and Cd#4). Cd#1 did not show any focal staining for SPARC. An identical immunolocalization protocol on TIC tumor transplants generated from the SPARC-transfected urospheres (TIC) showed focal expression of the SPARC protein in all 4 TIC tumor transplants with SPARC staining representing between 5 to 20% of the total tumor mass (Fig 4G and 4H, focal staining illustrated for As#3-SPARC and Cd#4-SPARC).

### Cell Morphology and Intracellular Localization of SPARC in Stably Transfected As^+3^-and Cd^+2^-Transformed UROtsa Cells

**Fig 6:**
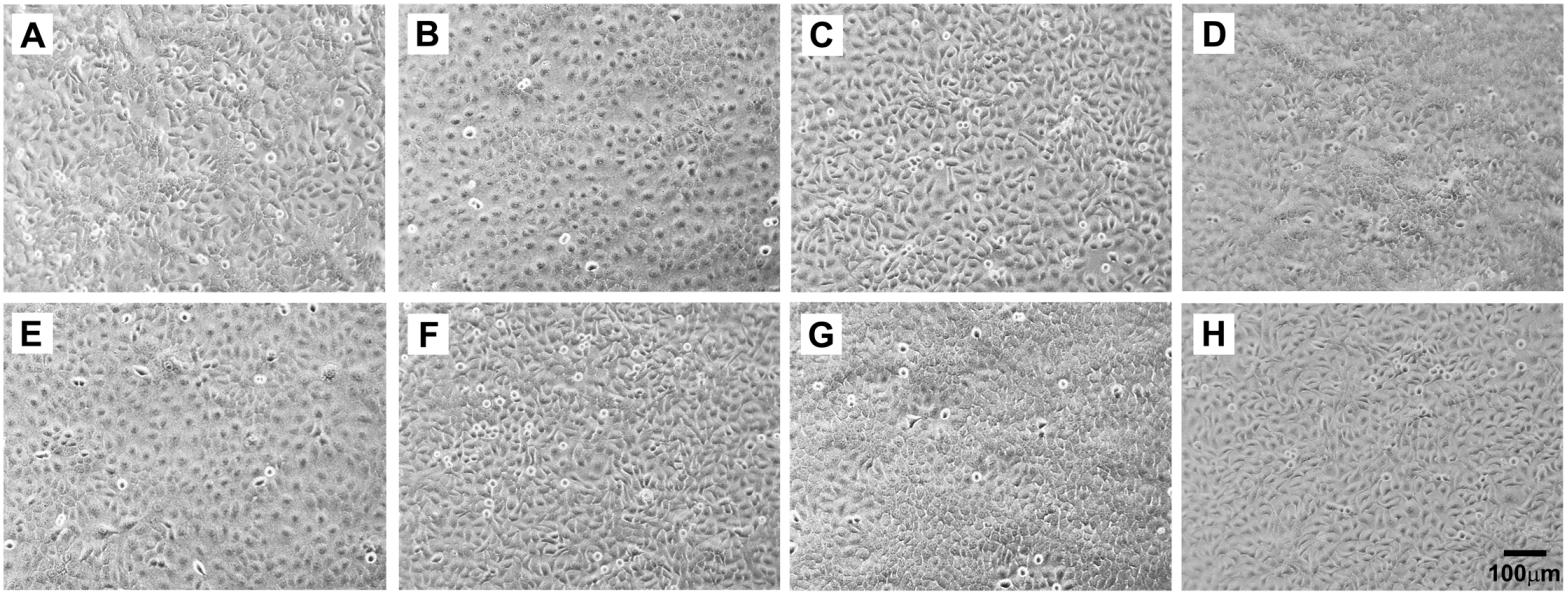
Phase contrast light microscopy of the SPARC-transfected and DEST-transfected UROtsa cell lines demonstrating epithelial morphology in all lines. (A) As#3-SPARC; (B) As#3-DEST; (C) As#6-SPARC; (D) As#6-DEST; (E) Cd#1-SPARC (F) Cd#1-DEST; (G) Cd#4-SPARC; and (H) Cd#4-DEST. The magnification of all images corresponds to the bar shown in H (100 μm).

The light level morphology of the SPARC transfected As^+3^-and Cd^+2^-transformed cell lines was determined and compared to that of the corresponding non-transfected As^+3^ and Cd^+2^ cell lines stably transfected with the blank vector (Fig 6A–6H). All the cell lines exhibited an epithelial morphology with no notable differences in morphology between those transfected with the SPARC containing vector and those with the vector without the SPARC ORF. In addition, the SPARC transfectants were also compared to the As^+3^-and Cd^+2^-transformed cell lines containing no expression vector and there were no differences in cell morphology (data not shown). Overall, there were no major changes in the light level morphology of the As^+3^-and Cd^+2^-transformed cell lines when they were stably transfected with the SPARC expression vector.

**Fig 7:**
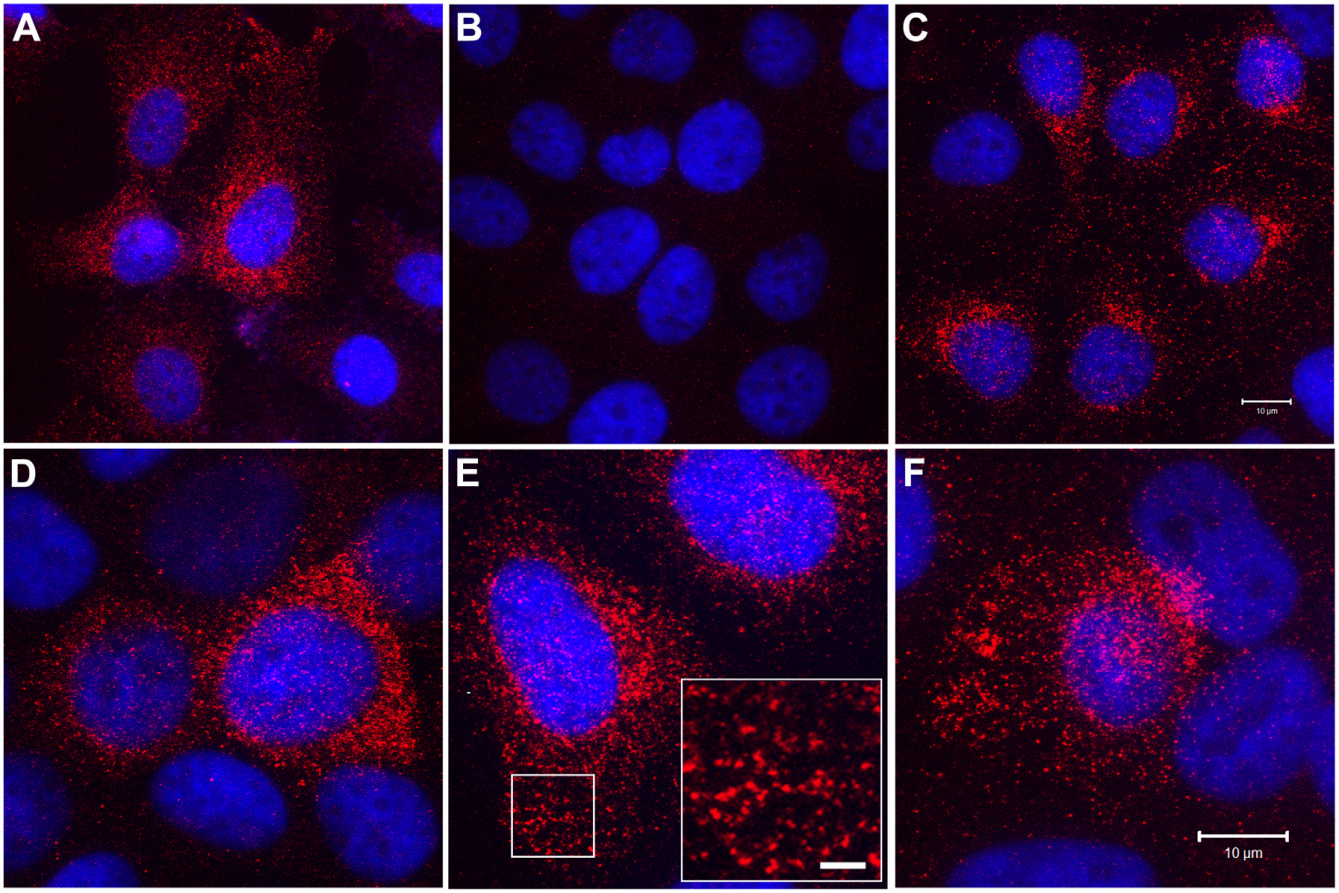
Intracellular localization of SPARC protein by immunofluorescent staining and confocal microscopy. SPARC (red) immunostaining as well as DAPI (blue) staining to identify all cells in the field is shown for the following UROtsa cell lines: (A) Parent; (B) Cd#4-DEST (blank vector); and (C) Cd#4-SPARC cells. Higher magnification images are shown for: (D) As#3-SPARC, (E) As#6-SPARC, and (F) Cd#1-SPARC cells. The inset shown in (E) for the As#6-SPARC cells shows a high magnification image of the region of cytoplasm indicated by the white box. This staining pattern is consistent with intracellular vesicles containing SPARC protein. Images in A-C correspond to bar in C (10μm), images in D-F correspond to the bar in F (10μm), and the bar shown in the inset corresponds to 2 μm.

The intracellular localization of the SPARC protein was also determined in the parental UROtsa cells and the SPARC-transfected As^+3^-and Cd^+2^-transformed cell lines (Fig 7A–7H). This analysis showed that the parental UROtsa cells had an intracellular expression of the SPARC protein, with only infrequent profiles of cells showing no SPARC immunoreactivity (Fig 7A). In contrast, the As^+3^-and Cd^+2^-transformed cell lines transfected with the blank vector showed no immunoreactivity for the SPARC protein (Fig 7B, As#6 shown for illustration). It was also demonstrated that the non-transfected As^+3^-and Cd^+2^-transformed cell lines had no immunoreactivity for the SPARC protein (data not shown). The majority of the cells in all four of the SPARC-transfected cell lines were immunoreactive for the SPARC protein, with only infrequent cell profiles negative for SPARC (Fig 7C–7F); similar to that noted for the parental UROtsa cell line. SPARC expression in the UROtsa parent and the SPARC transfected cell lines was localized throughout the cytoplasm and appeared as distinct vesicles (Fig 7E, As#6-SPARC for illustration). These studies show that the localization of SPARC in the transfectants was very similar to that seen in the parental UROtsa cells, suggesting that cells transformed by As^+3^ and Cd^+2^ do not alter the localization of SPARC when it is stably transfected back into these cell lines.

### Effect of SPARC Expression on Cell Growth, Migration and Invasion

The doubling times of the SPARC transfected As^+3^-and Cd^+2^-transformed cell lines were compared to the identical cell lines transfected with the expression vector without the SPARC ORF. It was demonstrated that SPARC expression had no effect on the doubling times of any of the transformed cell lines (Table 1). The doubling times of all the SPARC transfected cell lines as well as the cell lines transfected with the blank vector were significantly reduced compared to the parental UROtsa cells which have a doubling time of 33.2 ± 0.8 h [29, 30].

**Table 1:** Doubling Times of transfected UROtsa Cell Lines.

Cell Lines	As#3	As#6	Cd#1	Cd#4
**Transformed**	33.3 ± 1.4 h	21.6 ± 1.6 h	27.8 ± 0.6 h	20.7 ± 1.1 h
**SPARC**	27.4 ± 1.0 h*	25.6 ± 0.5 h*	23.8 ± 0.6 h*	22.0 ± 0.6 h
**DEST**	27.1 ± 0.5 h*	23.6 ± 0.7 h	22.7 ± 0.8 h*	23.5 ± 0.4 h*

The doubling times for the transformed cell lines (As#3, As#6, Cd#1, Cd#4) were previously described by Cao et al. 2010 [29] and Somji et al. 2010 [30].

*Denotes a statically significant difference compared to the non-transfected UROtsa cell line (p< 0.05).

**Fig 8:**
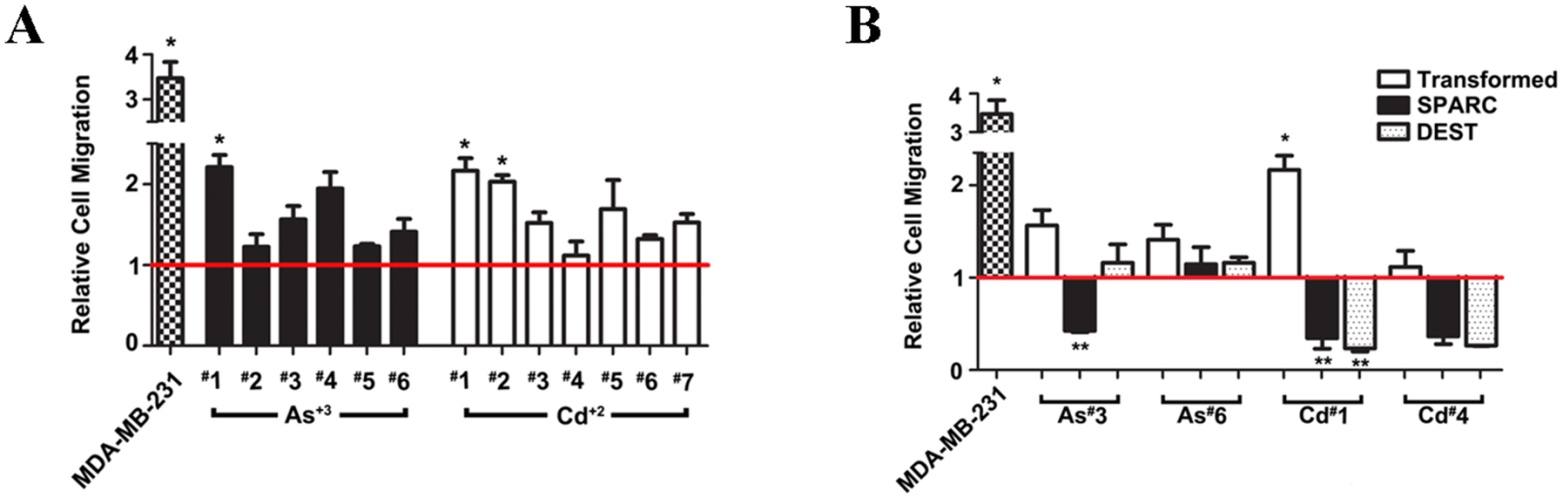
Relative cell chemotaxis migration of MDA-MB-231 and UROtsa cells transformed by As+3 or Cd+2 compared to the parent UROtsa cells. The red horizontal line at 1, represents the UROtsa parent cells. *Denotes a statistically significant difference from the UROtsa parent cells (p < 0.05). (B). Relative cell migration of MDA-MB-231, non-transfected UROtsa, SPARC-transfected, and blank vector (DEST) cells lines compared to the parent UROtsa cells. The red horizontal line at 1, represents the UROtsa parent cells. *Denotes a statistically significant difference from the UROtsa parent cells; ** denotes statistically significant difference from the non-transfected UROtsa counterpart (p < 0.05).

**Fig 9:**
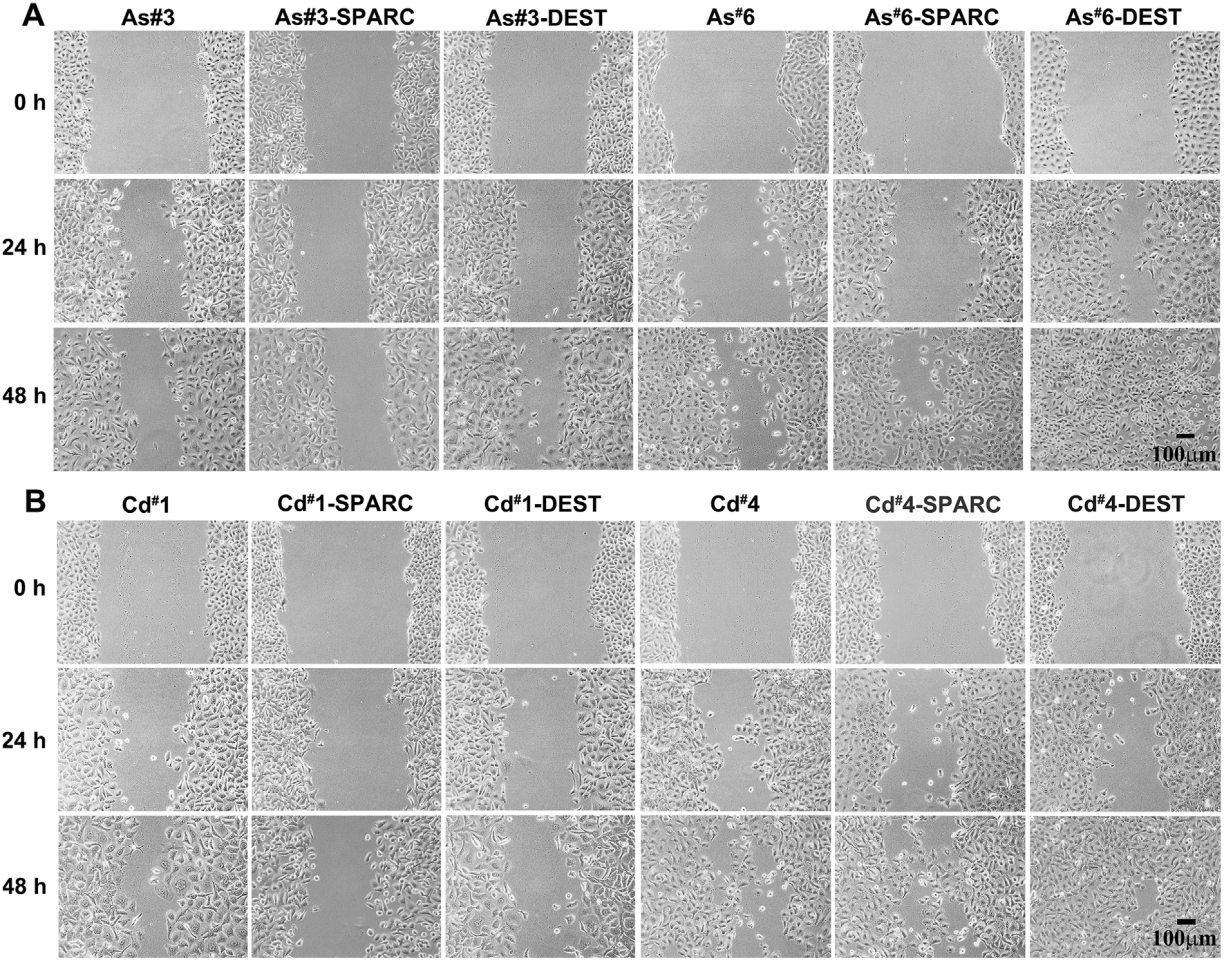
Wound healing assay of UROtsa As ^+3^ and Cd ^+2^ transformed cells transfected with SPARC or blank vector (DEST) and their non-transfected counterparts. Wound closing ability of each cell line was analyzed at 0, 24, and 48 h. Cells had been pretreated with MMC for 2 h prior to wounding and were shown to have no measurable growth during the 48 h period (data not shown). Panel (A) shows the As^+3^ cell lines while panel (B) features the Cd^+2^ transformed cell lines. The magnification of all images in a panel corresponds to bar in last image in the series: bar = 100 μm.

The effect of SPARC expression on cell migration was also determined on the SPARC transfected cell lines. However, for this analysis it was first necessary to determine the effect of As^+3^ and Cd^+2^ transformation on cell migration for the original cell lines since this has not been described previously. Cell migration was determined by the trans-well migration assay as well as the wound/scratch assay. For the trans-well migration assay, cell migration by chemotaxis was determined on the 6 independent isolates of the As^+3^-transformed UROtsa cells and the 7 independent isolates of the Cd^+2^-transformed cells. Chemotaxis of the transformed cell lines was compared to the UROtsa parental cell line and the well-characterized MDA-MB-231 breast cancer cell line. The results of this analysis showed that 2 of the As^+3^-transformed cell lines (#1 and #4) and 2 of the Cd^+2^-transformed cell lines (#1 and #2) showed significant increases in chemotaxis compared to the parental UROtsa cells (Fig 8A). The majority of the other As^+3^-and Cd^+2^-transformed lines also showed a trend for increased chemotaxis compared to the parental control, but the changes were not statistically significant. The effect of SPARC expression on chemotaxis was determined by comparing the chemotactic ability of the SPARC transfected cell lines to the corresponding non-transfected transformed cell line (Fig 8B). The results of this analysis showed that SPARC expression caused a significant decrease in chemotaxis for 3 of the 4 SPARC transfected cell lines (As#3, Cd#1, Cd#4) while the other cell line showed no significant differences between the SPARC transfected cell line and it non-transfected counterpart (As#6). A “scratch assay” was also used to determine the ability of the As^+3^-and Cd^+2^-transformed cell lines and their SPARC transfected counterparts to migrate and close a wound over a 48 h period. The results showed that the expression of SPARC had no effect on the ability of the As^+3^-and Cd^+2^-transformed cells to migrate and close the wounded area of the monolayer (Fig 9).

**Fig 10:**
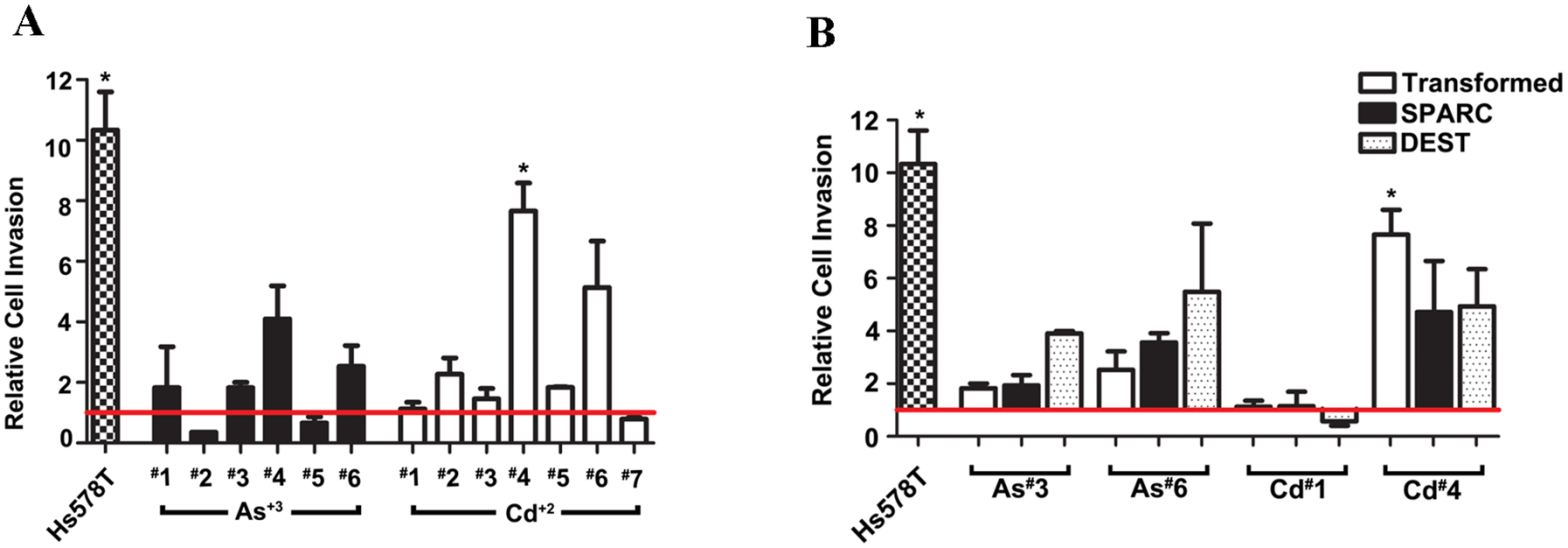
Relative cell invasion of Hs578T, non-transfected UROtsa, SPARC-transfected, and blank vector (DEST) cells lines compared to the parent UROtsa cells White graph bars represent the As^+3^ and Cd^+2^ transformed cell lines, the black graph bars represents SPARC transfected cell lines, while dotted bars represents blank vector transfected cell lines. Red horizontal line at 1 indicates the level of invasion of the UROtsa parent cells. *Denotes a statistically significant difference from the UROtsa parent cells (p < 0.05).

The effect of SPARC expression on cell invasion was also determined on the SPARC transfected cell lines. The effect of As^+3^ and Cd^+2^ transformation on the ability of the cells to invade was first determined on the original cell lines. This analysis showed that only 1 of the 6 As^+3^-transformed cell lines (#4) and 2 of the 7 Cd^+2^-transformed cell lines (#4, #6) had rates of invasion greater than that of the parental UROtsa cells (Fig 10A). The analysis also showed that the stable transfection and expression of SPARC in the 2 As^+3^-and 2 Cd^+2^-transformed cell lines had no effect on the ability on the cells to invade an artificial matrix (Fig 10B). The Hs578t cell line was used as a positive control.

## Discussion

A previous study from this laboratory demonstrated that SPARC expression was down-regulated to background levels in As^+3^-and Cd^+2^-transformed UROtsa cells compared to parental cells [31]. In addition, it was shown that SPARC was down-regulated to background levels in the malignant epithelial component of transplanted tumors derived from these cell lines, but that the murine stromal cells recruited to these tumors was highly reactive for SPARC. The absence of SPARC expression in the As^+3^-and Cd^+2^-transformed UROtsa cell lines afforded the opportunity to stably transfect the malignantly transformed cell lines with a SPARC expression vector to determine the effect of SPARC expression on the properties of the transformed cells. The results from stable transfection with the SPARC expression vector demonstrated that the 2 As^+3^-and 2 Cd^+2^-transformed cell lines had regained the *in vitro* expression of SPARC mRNA and protein at levels similar to or exceeding those of parental UROtsa cells. The intracellular localization of the SPARC protein in the SPARC-transfected As^+3^-and Cd^+2^-transformed cell lines was similar to the parental UROtsa cells, with SPARC expression localized throughout the cytoplasm and associated with distinct vesicles. This localization is consistent with SPARC being a secreted matricellular protein that regulates cell-matrix interactions and tissue remodeling through the binding of collagen and other extracellular matrix proteins [2, 3]. The intracellular localization of SPARC also demonstrated that there were only rare profiles (< 10%) of UROtsa parental cells and As^+3^-and Cd^+2^-transformed cells transfected with SPARC that did not express the SPARC protein. Cultures of the As^+3^-and Cd^+2^-transformed cell lines transfected with SPARC were also shown to secrete the SPARC protein into the growth media similar to that of the parental UROtsa cells. The finding that the SPARC protein was actively secreted under cell culture conditions makes it difficult to correlate levels of SPARC mRNA in the cell lines with intracellular levels of the SPARC protein, since SPARC protein expression would be the balance of the intracellular pool and that secreted by the cells. These *in vitro* properties of the SPARC transfected As^+3^ and Cd^+2^ cells are highlighted to show their similarity to SPARC expression in the parental UROtsa cell line. The similarity in SPARC expression between the parental UROtsa cells and the SPARC-transfected As^+3^-and Cd^+2^-cell lines provides important evidence that the SPARC gene is appropriately expressed in the transfected cell lines, including secretion into the culture media.

This is an important observation since the malignant epithelial component of the tumor transplants generated from the SPARC-transfected As^+3^-and Cd^+2^-transformed cell lines showed a low level of expression of SPARC mRNA and no expression of the SPARC protein by western blotting. The lack of expression of the SPARC protein in the malignant urothelial component of the transplanted tumors was surprising since a high percentage of the SPARC-transfected As^+3^-and Cd^+2^-transformed cell lines demonstrated the *in vitro* expression of SPARC. The low level of expression of the SPARC protein in lysates from the tumor transplants represents the average expression of SPARC within the entire tumor population, leaving the possibility of some focal expression of SPARC within the malignant urothelial component of the tumor. However, immunohistochemistry visualizes each individual cell and this technique complimented that of western blotting by demonstrating a low level of focal expression (less than 10%) in the tumor transplant generated from one SPARC-transfected cell line (As#3). Tumor generated from the other 3 (As#6, Cd#1 and Cd#7) SPARC-transfected cell lines showed no focal expression of SPARC. The combination of real time PCR, western blotting and immunohistochemistry convincingly demonstrated a complete suppression of SPARC expression in tumors generated from 3 of the 4 SPARC-transfected As^+3^-and Cd^+2^-transformed cell lines and only a low amount of focal expression of SPARC in the fourth cell line.

One possible explanation for the finding that SPARC expression was suppressed in the tumor transplants from the SPARC-transfected As^+3^-and Cd^+2^-transformed cell lines is that the TICs responsible for tumor formation did not express SPARC. This is a possible explanation since it is now generally accepted that only a sub-population of cells within a transformed cell line are capable of initiating a tumor transplant. A method has been developed to isolate a putative TIC population from such cultures by subculture of the cell line on non-adherent plastic and isolation of a population of cell spheroids. These spheroids are putative TICs and are associated with enhanced expression of aldehyde dehydrogenase 1A1 (ALDH1A1). The method has become standard for many cancers and has been employed to isolate TICs from the urothelial cancer cell lines, HTB-2, 4 and 9 [32, 33]. A necessary initial goal in the present study was to demonstrate that spheroids (urospheres) could be isolated from the non-SPARC transfected As^+3^-and Cd^+2^-transformed cell lines and that urospheres could initiate a tumor in immune-compromised mice. It was demonstrated that the urospheres isolated from the 2 As^+3^ (As#3 and As#6) and 2 Cd^+2^(Cd#1 and Cd#4)—transformed cell lines could initiate tumors in immune-compromise mice and that the tumors recapitulated the histology of the tumors produced when the parent cultures were inoculated into immune-compromised mice [28–30]. It was also shown that the urospheres had an elevated expression of ALDH1A1 compared to the parent cultures. The finding that the urospheres were capable of tumor initiation is consistent with their definition as a population of tumor initiating cells (TICs). This finding will allow the elucidation of the molecular signature of TICs isolated from an urothelial cell line transformed by As^+3^ and Cd^+2^, two common environmental pollutants.

An identical procedure was employed to isolate urospheres from the SPARC-transfected As^+3^-and Cd^+2^-transformed cell lines. Total RNA was isolated from the urospheres representing the 2 As^+3^ and 2 Cd^+2^ SPARC-transfected cell lines and used to determine the level of ALDH1A1 and SPARC mRNA expression. The level of ALDH1A1 mRNA was elevated compared to the parent cultures and similar to the levels found in urospheres from the non-SPARC transfected As^+3^-and Cd^+2^-transformed cell lines. In marked contrast, the level of SPARC mRNA in the urospheres was reduced to near background levels of expression compared to the level present in the SPARC-transfected As^+3^-and Cd^+2^-transformed cell lines. The finding that the urospheres from the SPARC transfected cells had a very low expression of SPARC mRNA could explain the lack of SPARC expression in the tumor transplants generated from the SPARC-transfected As^+3^-and Cd^+2^-transformed cell lines. To confirm this notion, the urospheres from the non-SPARC transfected and SPARC-transfected As^+3^-and Cd^+2^-transformed cell lines were inoculated into immune compromised mice, with the assumpion being that there would be no expression of SPARC. However, this was found not to be the case. An immunohistochemical analysis for SPARC expression demonstrated focal staining for SPARC in 3 of the 4 tumors derived from urospheres isolated from the non-SPARC transfected As^+3^-and Cd^+2^-transformed cell lines. Similarly, immunostaining for SPARC demonstrated focal staining for SPARC in all 4 tumors derived from urospheres isolated from the SPARC transfected As^+3^-and Cd^+2^-transformed cell lines. The focal expression of SPARC in the tumor transplants was in agreement with the finding that SPARC mRNA was expressed in these tumors; however, western blotting did not detect the SPARC protein. The lack of SPARC protein on western analysis can be explained by the fact that only approximately 10% of the transplanted tumors expressed SPARC and that real time PCR is a far more sensitive method to detect mRNA expression. In addition, focal expression of a protein within a tumor is subject to sampling errors depending on the area of tumor dissected for lysate preparation. These results show that TICs isolated from both SPARC transfected and non-SPARC transfected As^+3^-and Cd^+2^-transformed cell lines can produce tumors with a low incidence of focal expression of the SPARC protein.

The results of this study show that TICs from the SPARC transfected As^+3^-and Cd^+2^-transformed cell lines are suppressed for their expression of SPARC mRNA, while the majority of cells in the overall culture from which the TICs arise are permissive for the expression of SPARC mRNA. That such a population exists in the overall culture of the As^+3^-and Cd^+2^-transformed cell lines is supported by the examination of the cells for the intracellular localization of SPARC. In this examination, it was noted that there were rare profiles of cells in each SPARC-transfected cell line that did not express SPARC and these were estimated to be less than 1 in every 10 cells. These rare cells that do not express SPARC, or a subset thereof, are presumably the cells comprising the population of TICs that initiated the tumor transplants. The laboratory has not yet determined the mechanism underlying the ability of the TICs from the SPARC-transfected As^+3^-and Cd^+2^-transformed cell lines to silence the expression of SPARC mRNA under cell culture conditions. One explanation that can be removed from consideration is that there can be no interaction of the TICs regulatory components with the regulatory regions of the SPARC gene, since these sequences were not present in the expression vector used for the stable transfection of the SPARC gene. This includes the promoter region, the 3' untranslated region and intron sequences. Less likely explanations, but not inconceivable, are that some property of the TICs does not allow the uptake and stable transfection of the expression vector harboring the SPARC gene or that some undefined elements present in the TICs silences the CMV promoter of the expression vector. One can speculate that the most likely explanation will involve the expression of one or more microRNAs, or other regulatory RNAs, in the TIC population that are able to prevent the expression and translation of SPARC mRNA. This mechanism would be consistent with the finding that the TICs had a near background expression of SPARC mRNA. In addition, no literature could be found which indicates how common or rare it is to find a gene, such as SPARC in the present case that is silenced in a TIC population when the majority of other cells in the culture can be stably transfected with an expression vector.

The involvement of a mechanism such as a regulatory RNA would also be consistent with the finding that TICs from the non-SPARC transfected and SPARC transfected As^+3^-and Cd^+2^-transformed cell lines were able to initiate tumors with the focal expression of SPARC. With the exception of the TIC-initiated tumor derived from one non-SPARC transfected cell line, the other 3 TIC-derived tumors all had a similar level of focal expression of the SPARC protein, showing moderate immunoreactivity in approximately 10% of the tumor. It is possible that the one tumor without SPARC is simply a sampling error. The finding that SPARC expression was similar in both the TIC-derived tumors from both the SPARC transfected and non-SPARC transfected suggests that the SPARC expression vector had no influence on the ability of the TIC-derived tumors to express the SPARC protein. This would suggest that the ability to initiate the focal expression of SPARC in the TIC-derived tumors is due to some unknown feature of the tumor environment. A change in tumor environment could be expected to alter the regulatory RNA profile of the TICs. What is unknown in the present study is if the focal expression of SPARC is a favorable sign of tumor differentiation or one of tumor progression. This is especially true for the SPARC protein since as detailed in the introduction it has been found to have contrasting properties and has been proposed to be a tumor suppressor or an oncogene depending on the tumor. Serial passage of the tumor transplants would be necessary to determine if the focal expression of SPARC is an overall feature of these tumors or an element lost upon serial passage.

An additional caveat in the present study is that the urospheres isolated from the non-transfected and SPARC-transfected As^+3^-and Cd^+2^-cells may only be indicative of one set of TICs that could exist in the cell culture and that can also form subcutaneous tumors in immune-compromised mice. The isolation of the TICs is dependent on a cell culture method and it is possible that another type of isolation could also identify a population of TIC that might have different properties. However, the correlation in the present study between SPARC expression in the TICs isolated from the cultured cells and that of the transplant tumors is noteworthy for one population.

The effect of SPARC transfection on cell growth, light level morphology, chemotaxis and invasion was determined on the overall cell population representing the 2 As^+3^-and 2 Cd^+2^- transformed cell lines. No effect of SPARC expression was noted for cell growth or the morphology of the cells. Three of the 4 SPARC transfected cell lines did show a reduction in chemotaxis when compared to their non-transfected counterparts. However, there was no effect of SPARC transfection on the ability of the transformed cells to close a wound that was generated using the “scratch assay”. The expression of SPARC also had no effect on the invasiveness of the transformed cell lines.

In conclusion, this study demonstrates that TICs can be isolated from cultures of UROtsa cells malignantly transformed by either As^+3^ or Cd^+2^. The study also shows that a mechanism exists for these transformed cells to suppress the expression of SPARC mRNA in TICs isolated from both, SPARC transfected and non-SPARC transfected As^+3^-and Cd^+2^-transformed cell lines. However, tumor formation in immune compromised mice demonstrated that the focal expression of SPARC can be regained following inoculation of TICs from both SPARC transfected and non- SPARC transfected As^+3^-and Cd^+2^-transformed cell lines.

## Supporting Information

S1 Table STR Profiling of the UROtsa cell line. (DOCX)

S2 Table SPARC Primers and ORF. (DOCX)

S3 Table Vector Copy Number. (DOCX)
