# Supplementary material for: SPARC Expression Is Selectively Suppressed in Tumor Initiating Urospheres Isolated from As+3- and Cd+2-Transformed Human Urothelial Cells (UROtsa) Stably Transfected with SPARC
Source: PLoS One. 2016 Jan 19;11(1):e0147362. doi: 10.1371/journal.pone.0147362 (PMC4718619; doi:10.1371/journal.pone.0147362)
Supplement: S1 Table — (DOCX) [file pone.0147362.s001.docx]

| **S1 Table.** | |
| --- | --- |
| Loci |  |
| AMEL | X, X |
| D5S818 | 12, 13 |
| D13S317 | 11, 13 |
| D7S820 | 11, 12 |
| D16S539 | 11, 12 |
| vWA | 16, 16 |
| TH01 | 6, 9.3 |
| TPOX | 8, 9 |
| CSF1PO | 11, 12 |

STR Profiling of the UROtsa Cell line
