# Supplementary material for: SPARC Expression Is Selectively Suppressed in Tumor Initiating Urospheres Isolated from As+3- and Cd+2-Transformed Human Urothelial Cells (UROtsa) Stably Transfected with SPARC
Source: PLoS One. 2016 Jan 19;11(1):e0147362. doi: 10.1371/journal.pone.0147362 (PMC4718619; doi:10.1371/journal.pone.0147362)
Supplement: S2 Table — (DOCX) [file pone.0147362.s002.docx]

**SPARC Open Reading Frame**

ATGAGGGCCTGGATCTTCTTTCTCCTTTGCCTGGCCGGGAGGGCCTTGGCAGCCCCTCAG

CAAGAAGCCCTGCCTGATGAGACAGAGGTGGTGGAAGAAACTGTGGCAGAGGTGACTGAG

GTATCTGTGGGAGCTAATCCTGTCCAGGTGGAAGTAGGAGAATTTGATGATGGTGCAGAG

GAAACCGAAGAGGAGGTGGTGGCGGAAAATCCCTGCCAGAACCACCACTGCAAACACGGC

AAGGTGTGCGAGCTGGATGAGAACAACACCCCCATGTGCGT**GTGCCAGGACCCCACCAGC**

**TGCCCAGCCCCCATTGGCGAGTTTGAGAAGGTGTGCAGCAATGACAACAAGACCTTCGAC**

**TCTTCCTGCCACTTCTTTG**CCACAAAGTGCACCCTGGAGGGCACCAAGAAGGGCCACAAG

CTCCACCTGGACTACATCGGGCCTTGCAAATACATCCCCCCTTGCCTGGACTCTGAGCTG

ACCGAATTCCCCCTGCGCATGCGGGACTGGCTCAAGAACGTCCTGGTCACCCTGTATGAG

AGGGATGAGGACAACAACCTTCTGACTGAGAAGCAGAAGCTGCGGGTGAAGAAGATCCAT

GAGAATGAGAAGCGCCTGGAGGCAGGAGACCACCCCGTGGAGCTGCTGGCCCGGGACTTC

GAGAAGAACTATAACATGTACATCTTCCCTGTACACTGGCAGTTCGGCCAGCTGGACCAG

CACCCCATTGACGGGTACCTCTCCCACACCGAGCTGGCTCCACTGCGTGCTCCCCTCATC

CCCATGGAGCATTGCACCACCCGCTTTTTCGAGACCTGTGACCTGGACAATGACAAGTAC

ATCGCCCTGGATGAGTGGGCCGGCTGCTTCGGCATCAAGCAGAAGGATATCGACAAGGAT

CTTGTGATCTAG

Yellow designates the 4^th^ exon

Blue Green designates the 5^th^ exon

Red designates SPARC primer binding region

**SPARC Primers**

QuantiTech Primers from Qiagen (QT00018620)

Details available at: <https://www.qiagen.com/us/shop/pcr/real-time-pcr-enzymes-and-kits/two-step-qrt-pcr/quantitect-primer-assays?catno=QT00018620#geneglobe>

Primer sequence: proprietary

Product Size: 60 bps

SPARC mRNA primer binding site: in 4^th^ and 5^th^ exon (designated above). The sequence in red is the most probable region assuming 60 bps.

**18S rRNA Primers**
Upper primer:  CGCCGCTAGAGGTGAAATTC
Lower primer: TTGGCAAATGCTTTCGCT
Product size of 62 bp

Annealing temperature: 65ºC
