# Supplementary material for: SPARC Expression Is Selectively Suppressed in Tumor Initiating Urospheres Isolated from As+3- and Cd+2-Transformed Human Urothelial Cells (UROtsa) Stably Transfected with SPARC
Source: PLoS One. 2016 Jan 19;11(1):e0147362. doi: 10.1371/journal.pone.0147362 (PMC4718619; doi:10.1371/journal.pone.0147362)
Supplement: S3 Table — (DOCX) [file pone.0147362.s003.docx]

**S3 Table**

**Vector Copy Number in Transfected Cell Lines versus cultured urospheres (TIC)***

|  | **Cell Lines** | | **TIC** | |
| --- | --- | --- | --- | --- |
|  | Copy Number | SE | Copy Number | SE |
| **As#3** | 5.67 | 0.209 | 3.74 | 0.07 |
| **As#6** | 1.38 | 0.035 | 1.97 | 0.083 |
| **Cd#1** | 2.99 | 0.146 | 1.86 | 0.022 |
| **Cd#4** | 1.13 | 0.0527 | 0.68 | 0.014 |

*Assessed with pPCR and expressed as copies per cell assuming 6.2 pg of

genomic DNA per cell.
